# Effect of a Point Mutation in *mprF* on Susceptibility to Daptomycin, Vancomycin, and Oxacillin in an MRSA Clinical Strain

**DOI:** 10.3389/fmicb.2018.01086

**Published:** 2018-05-25

**Authors:** Feng-Jui Chen, Tsai-Ling Lauderdale, Chen-Hsiang Lee, Yu-Chieh Hsu, I-Wen Huang, Pei-Chi Hsu, Chung-Shi Yang

**Affiliations:** ^1^National Institute of Infectious Diseases and Vaccinology, National Health Research Institutes, Zhunan, Taiwan; ^2^Division of Infectious Diseases, Kaohsiung Chang Gung Memorial Hospital, College of Medicine, Chang Gung University, Kaohsiung, Taiwan; ^3^Institute of Biomedical Engineering and Nanomedicine, National Health Research Institutes, Zhunan, Taiwan

**Keywords:** MRSA, evolution, drug resistance, daptomycin, vancomycin, oxacillin

## Abstract

We previously reported the sequential recovery of daptomycin-nonsusceptible MRSA clinical isolates with an L431F substitution in the MprF protein. The aim of the present study is to determine the effect of this mutation by replacing the *mprF* gene on the chromosome of a daptomycin-susceptible progenitor strain, CGK5, to obtain CGK5mut having the L431F MprF mutation. Compared to CGK5, the daptomycin and vancomycin MICs of CGK5mut increased from 0.5 to 3 μg/ml and from 1.5 to 3 μg/ml, respectively; however, its oxacillin MIC decreased from 128 to 1 μg/ml in medium without added 2% NaCl. The expression levels of *vraSR* and several other cell-wall synthesis-related genes were significantly increased in CGK5mut, and the mutant also had significantly reduced negative cell membrane charge, thicker cell wall, and longer doubling time. These features were abolished in the reverse mutant carrying F431L MprF, confirming the pleiotropic effects of the L431F MprF mutation. We believe that this is the first work that shows a single MprF missense mutation can lead to not only changes in the cell membrane but also increased expression of *vraSR* and subsequently increased resistance to daptomycin and vancomycin while simultaneously conferring increased susceptibility to oxacillin in an isogenic MRSA strain.

## Introduction

Daptomycin, a cyclic lipopeptide antibiotic, is one of the last-line agents for the treatment of certain severe multidrug-resistant *Staphylococcus aureus* infections, including those caused by methicillin-resistant *S. aureus* (MRSA) (Enoch et al., 2007). Daptomycin functions by inserting itself into the bacterial cell membrane in a calcium-dependent manner to cause membrane depolarization, leading to cell death (Enoch et al., 2007; Baltz, 2009). Daptomycin-nonsusceptible (DAP-NS) MRSA isolates, although still uncommon, have emerged during daptomycin treatment of patients (Lee et al., 2010; Boyle-Vavra et al., 2011). DAP-NS MRSA mutants have also been generated in the laboratory by serial passage of isolates in sublethal concentrations of daptomycin (Enoch et al., 2007; Camargo et al., 2008; Mishra et al., 2009, 2012; Rubio et al., 2012).

The exact mechanisms giving rise to daptomycin-non-susceptibility in *S. aureus* are not fully elucidated but appear to involve diverse genetic events and several genetic loci, including *mprF*, *yycG* (*walK*), *vraSR*, *tagA*, and *dltABCD* (Friedman et al., 2006; Baltz, 2009; Bertsche et al., 2011; Song et al., 2013). These loci are all part of the cell wall stimulon in *S. aureus* and include genes encoding proteins involved in the production of membrane phospholipids. The *mprF* (multiple peptide resistance factor) gene seems to be especially critical, as *mprF* mutations are the most frequently reported genetic lesions in DAP-NS MRSA isolates (Friedman et al., 2006; Lee et al., 2010; Boyle-Vavra et al., 2011; Mehta et al., 2012a). MprF is a bi-functional membrane protein with lysylphosphatidylglycerol (LPG) synthase and flippase activities (Peschel et al., 2001; Ernst et al., 2009; Ernst and Peschel, 2011). Different point mutations in *mprF* have been associated with elevated LPG synthesis. This results in increased amounts of LPG relative to phosphatidylglycerol (PG) on the outer leaflet of the cytoplasmic membrane and an accompanying reduction in cell membrane negative charge (Baltz, 2009; Rubio et al., 2012).

A feature that has been seen in both clinical and laboratory-generated DAP-NS MRSA isolates is a concomitant vancomycin intermediate or heterogeneous intermediate resistance (VISA or hVISA) phenotype (Camargo et al., 2008; Mishra et al., 2009); VISA has moderate resistance to vancomycin and hVISA has varying sub-population of cells resistant to vancomycin, thus exhibit mixed susceptibility to vancomycin. Mutations in the *vraSR*, *graSR*, or *walKR* (*yycGF*) two-component systems have been associated with the VISA/hVISA phenotype (Howden et al., 2010). Among these 3 two-component systems, the VraSR system is particularly important in maintaining cell wall integrity. It serves as a sentinel in response to cell wall damage by positively regulating a unique set of genes involved in cell wall synthesis, resulting in the generation of a resistant phenotype (Kuroda et al., 2003; Gardete et al., 2006; McAleese et al., 2006). Another unusual feature is the so-called “seesaw” effect on β-lactam susceptibility, wherein DAP-NS isolates exhibit reduced β-lactam MICs (Mishra et al., 2009; Lee et al., 2010; Yang et al., 2010; Mehta et al., 2012a). However, these phenomena are not observed in all DAP-NS MRSA isolates.

Our previous study of eight sequential clinical MRSA isolates from a patient with persistent bacteremia revealed an L431F amino acid substitution in the MprF protein of DAP-NS isolates (Lee et al., 2010). Since this mutation had not been reported previously, we undertook the present study to determine the effect of this mutation on the cellular response to daptomycin. To eliminate the possibility of unknown genetic changes that might have occurred in paired clinical strains, we used a base-substitution method to replace a single nucleotide (from CTT to TTT) within the chromosomal *mprF* gene of the daptomycin-susceptible (DAP-S) progenitor of the DAP-NS strains. To confirm the results obtained by this single amino acid exchange (L431F) in MprF, we also constructed a reverse mutant carrying F431L MprF as well as a silent EcoRV site. Our study demonstrated that this single amino acid change (L431F) confers increased resistance to both daptomycin and vancomycin, with a concurrent decrease in oxacillin MIC. The phenotype and genetic factors associated with these changes were investigated.

## Materials and Methods

### Bacterial Strains

The bacterial strains, plasmids, and primers used are listed in **Tables 1**, **2**. Unless stated otherwise, Luria-Bertani (LB) broth and plates were used for growth of *Escherichia coli* and *S. aureus* at 37°C. *E. coli* strain XL10-Gold (Stratagene, La Jolla, CA, United States) and GeneHogs^®^ (Invitrogen, Carlsbad, CA, United States) were used for cloning. *S. aureus* cells were transformed by electroporation, as described previously (Schenk and Laddaga, 1992). Ampicillin (100 μg/ml), chloramphenicol (5 μg/ml), erythromycin (5 μg/ml), spectinomycin (100 μg/ml), and tetracycline (5 μg/ml) were used for plasmid selection in *E. coli* and *S. aureus*.

**Table 1:** Bacterial strains and plasmids used in this study.

Strain/plasmid	Description	Source or Reference
**Strains**		
*E. coli*		
XL10-Gold	Ultra-competent cell for site-directed mutagenesis	Stratagene
Genehogs	Electrocompetent cells	Invitrogen
*S. aureus*		
RN4220	Restriction-deficient derivative of 8325-4	Novick, 1991
RN6911	RN6390B *agr*::*tetM* (*agr*-null)	Novick et al., 1993
Z172	Clinical VISA isolate with *spc* gene	Chen et al., 2013
CGK5	Daptomycin-susceptible MRSA	Lee et al., 2010
CGK5mut	MprF L431F derivative of CGK5	This study
CGK5mutR	Reversed derivative of CGK5mut with MprF containing F431L and a new EcoRV site	This study
CGK6	Daptomycin-non-susceptible MRSA	Lee et al., 2010
*E. faecium*		
2V076	Clinical isolate with *aadE*-*sat4*-*aphA*-3 gene cluster	This study
**Plasmids**		
pMAD	pE194^ts^ derivative for gene replacement in Gram-positive bacteria	Arnaud et al., 2004
pMAD-SAT4-tetM	Modified pMAD with *sat4* and *tetM* markers	This study
pMprFmut	The *mprF* fragment amplified from CGK6 and cloned into pMAD-SAT4-tetM for allelic exchange in CGK5	This study
pMprF5	The *mprF* fragment amplified from CGK5 and cloned into pMAD-SAT4-tetM for site-directed mutagenesis	This study
pMprFmutR	A silent EcoRV site was introduced into the middle of the *mprF* fragment amplified from pMprF5 and cloned into pMAD-SAT4-tetM for allelic exchange in CGK5mut	This study
pluxT2	pSK5630 derivative containing *luxABCDE* with T2 terminator	Chen et al., 2014
pluxT2-SPC	pluxT2 with *spc* marker	This study

*^ts^Stands for thermosensitive.*

**Table 2:** Primers used in this study.

Primers	Nucleotide sequence (5′–3′)^a^
**Cloning of new pMAD**
*bgaB*new-F	CGGGATCCAGGAATTCGCTCCCGGGCATGCCATGGGTCTAGTTAATGTGTAACGTAACA
*bgaB*new-R	ACGCGTCGACGTAAGGCCTTCACTAAACCTTCCCGGCTTC
*sat4*-F	P-AGAGAGGCGGGAACAGTG
*sat4*-R	ACGCGTCGACTGCAGGCCTTCAGATCTAAGACGAACTCCAATTCACT
*tetM*-F	P-GGAGATTCCTTTACAAATATG
*tetM*-R	ACGCGTCGACGTAAGGCCTATAACAACATAAAACGCACTA
**Cloning of *mprF* gene for allelic exchange**
*mprF*-F	CGGGATCCTAGAATTGATGTGAAAAAATGA
*mprF*-R	TCCCCCGGGCGCATCAGGCATAACTGTATA
**Site-directed mutagenesis**
*mprF*(EcoRV)-F	P-AT**C**ATTGCTAAAATTCCATCATTGTC
*mprF*(EcoRV)-R	ATCCTTTTGATAAGACATTAAAA
**Real-time qPCR**
*mprF*-QF	TCATTATTGCTGCATTATCTGGA
*mprF*-QR	TTTTCCTCAGGGACACCTAAAG
*vraSR*-QF	GCCAGATTCAGGTACACG
*vraSR*-QR	TCTGAGTCGTCGCTTC
*fmtA*-QF	AAAACATCTAAGCCTATCCCATTG
*fmtA*-QR	TTTGAATCGCTTTAACTGCTTGAT
*murZ*-QF	AAAATAAGAGGTGGACGCACA
*murZ*-QR	ACTGTTTTTCGCGCCACT
*pbp2*-QF	TCGGTGCAATTGGTAAGAACT
*pbp2*-QR	TTAATGTTGAGGCACCTTCAGA
*sgtB*-QF	TAGCGACAGAGATGTGC
*sgtB*-QR	TTGTGACATAGCCTGTTG
*tagA*-QF	AATAAATCAAGCGAGCTATATTGTTG
*tagA*-QR	ACGATGCGAAGCTTTGACTAC
*gyrB*-QF	CGTTAATTGAAGCAGGCTATGTG
*gyrB*-QR	TGGTGTTGGATTCAATTCAGATT
**Cloning of promoter-reporter**
*spc*-F	P-AAAGTTCTCGTTCGGAGG
*spc*-R	TCCCCCGGGAAAGTAAGCACCTGTTATTGC
P*mprF*-F	CGGGATCCGAAAATAAAAACAAGTGGTAT
P*mprF*-R	ACGCGTCGACTTAACTTCCTGATTCATTT
P*vraSR*-F	CGGGATCCCGTTTATCTCATCAAATG
P*vraSR*-R	ACGCGTCGACTAGTTCATAACTATCACCTTT

*^a^Restriction enzyme sites are underlined. P represents phosphorylation. Boldface type indicates a mutant nucleotide.*

### Construction of MprF Mutant Derivatives

To add new selection markers and cloning sites, the *bgaB* cassette located between the HindIII sites was removed from pMAD by digesting with HindIII, and religated. A modified *bgaB*, with extra restriction sites, was cloned by PCR (using primers *bgaBnew*-F and *bgaBnew*-R) from the original pMAD into the pMAD lacking the *bgaB* cassette to generate new pMAD. Two selection markers, as *sat4* and *tetM* cassettes, were incorporated by cloning PCR fragments of the *sat4* and *tetM* genes from the chromosomal DNA of 2V076 and RN6911, respectively, into the StuI-SalI sites of the new pMAD using primers *sat4*-F and *sat4*-R, and *tetM*-F and *tetM*-R to produce pMAD-SAT4-tetM, which confers resistance to nourseothricin and tetracycline. To assess the effect of MprF L431F on daptomycin non-susceptibility, an *mprF*-bearing fragment from CGK6 (the first DAP-NS isolate containing the MprF F431 mutant) was amplified by PCR using the primers *mprF*-F and *mprF*-R, and ligated into the BamHI-SmaI sites of pMAD-SAT4-tetM. This recombinant plasmid, pMprFmut, was used as the allelic exchange vector for mutation of the *mprF* gene in CGK5 to create CGK5mut. To validate the phenotypes in CGK5mut, the mutant strain was reverted to wild type by allelic exchange again. The *mprF* fragment was amplified from CGK5 and then cloned into pMAD-SAT4-tetM. This recombinant plasmid, pMprF5, was used as the template DNA for site-directed mutagenesis to introduce a new EcoRV site^1^ into the complementation construct (and without altering the coding sequence) to allow it to be differentiated from the CGK5 parental strain. After PCR using the mutant primers *mprF*(EcoRV)-F and *mprF*(EcoRV)-R, the *mprF* reverse mutant was treated with DpnI, ligated into a circle with T4 DNA ligase, and then used as the template DNA. The entire *mprF* gene was then cloned from the mutated plasmid into the pMAD-SAT4-tetM vector again to eliminate potential mutation of the vector sequence. The constructs were verified by restriction analysis and DNA sequencing. Sequencing was performed at the DNA Sequencing Core Lab of our institutes.

The allele replacement procedure was applied to create a single base replacement in the *mprF* gene of CGK5, as described previously (Arnaud et al., 2004). Briefly, the pMprFmut plasmid was electroporated into *S. aureus* strain RN4220, and then electroporated into CGK5. Transformants were selected at 30°C on LB plates containing tetracycline and X-Gal (150 μg/ml). One blue colony was inoculated in Trypticase Soy broth (TSB) containing tetracycline and incubated with shaking for 2 h at 30°C followed by 6 h at 43°C, serial diluted then plated on Trypticase Soy agar (TSA) plates containing tetracycline and X-Gal and incubated at 43°C overnight to obtain light blue colonies caused by a single crossover event. One light blue colony was inoculated in TSB without antibiotic and incubated with shaking at 30°C overnight, then diluted 1:100 and incubated at 43°C for 6 h; serial dilutions were plated on TSA plates in the absence of antibiotics and incubated at 37°C overnight. Several white colonies were selected to verify for tetracycline sensitivity, which indicates loss of the integrated vector resulting from a double crossover event. To confirm the double crossover, PCR amplifications were performed with multiplex primers hybridizing outside and inside of the *mprF* gene and vector sequences. A colony with a single crossover was used as a negative control. The resulting mutant was verified by DNA sequencing, including the adjacent region of the *mprF* gene. The complementation construct (CGK5mutR) was constructed by the same procedure.

### Antimicrobial Susceptibility Testing

The MICs of daptomycin, oxacillin and vancomycin were determined by Etest^®^ (bioMérieux SA, Marcy l’Étoile, France) following the manufacturer’s instructions and using Mueller Hinton II agar (MHA) (Becton Dickinson, Cockeysville, MD, United States). The daptomycin Etest strips were overlaid with 40 μg/ml of calcium (Package insert) and the MHA contained 2.9–5.9 μg/ml of calcium. The MICs of oxacillin were also determined by broth microdilution (BMD) (CLSI, 2013). The BMD method was performed in Mueller Hinton II broth (MHB) (Becton Dickinson) with and without 2% NaCl from an inoculum of 5 × 10^5^ CFU/ml, and the MIC was read after incubation at 35°C for 24 h. *S. aureus* ATCC 29213 and *Enterococcus faecalis* ATCC 29212 were used as quality control organisms for Etest. *S. aureus* ATCC 29213 and ATCC 43300 were used as quality control organisms for BMD.

### Population Analysis Profiles

A population analysis profile (PAP) for vancomycin was performed on CGK5 and CGK5 mutant derivatives following protocols previously described (Hiramatsu et al., 1997; Howden et al., 2006). Briefly, overnight cultures of test isolates were serially diluted in TSB and inoculated onto brain–heart infusion (BHI) agar plates containing 0–8 μg/ml vancomycin. After 48 h incubation at 35°C, colonies were counted and plotted. Mu3 (ATCC 700698) and N315 *S. aureus* strains were tested in parallel as hVISA positive and negative controls, respectively. The area under the curve (AUC) values of the test strains were compared to that of Mu3.

### Cell Wall Thickness

Bacterial cells for transmission electron microscopy were prepared following previously described protocols (Hanaki et al., 1998). Photographic images were obtained at a final magnification of 15,000× using a Hitachi H-7650 microscope (Hitachi High-Technologies Corporation, Tokyo, Japan). Fifty measurements of equatorially cut cells were taken for the calculation of cell wall thickness, and results were expressed as mean ± SD following previously described protocols (Cui et al., 2000).

### Growth Rate

Overnight fresh cultures of bacteria were adjusted in 0.85% NaCl to 0.5 McFarland turbidity, then diluted 1:200 in MHB to obtain 5 × 10^5^ CFU/ml starting inoculum. The inoculum was dispensed at 120 μl per well in triplicates into a 100-well plate and incubated at 37°C in Bioscreen C MBR (Oy Growth Curves Ab Ltd, Helsinki, Poland) (Richardson et al., 2008). Triplicate medium-only blank wells were included in each plate. The OD_600_ of each well was read every 30 min for 24 h. The average OD of the blank wells was subtracted from the average of the triplicate test wells at each time point and plotted. Doubling times were calculated using the exponential growth phase following a previously described protocol (Cui et al., 2003). To verify the OD measurements, the CFU counts were checked by the shaker flask method.

### Cytochrome *c* Binding Assay

Cytochrome *c* binding assay was performed following an approach similar to that of Mukhopadhyay et al. (2007) with slight modification. Briefly, bacteria grown overnight at 35°C were harvested and washed twice with 20 mM MOPS buffer (pH 7.0) and resuspended in the same buffer to a final OD_578_ of 7. Cytochrome *c* (Sigma Chemicals, St. Louis, MO, United States) was prepared in the same buffer. The bacterial suspension was incubated with 1 mg/ml cytochrome *c* for 10 min, and then centrifuged at 3000 *g* at 4°C for 10 min. The supernatant containing unbound cytochrome *c* was collected and measured spectrophotometrically at OD_530_. The cytochrome *c* was serially diluted (0.1–0.6 mg/ml) to create a standard curve to measure the cytochrome *c* concentration of the supernatant.

### Western Blot Analysis for PBP2a Detection

Detailed descriptions of mouse monoclonal anti-PBP2a antibody (2F6F) production and detection of PBP2a were presented previously (Chen et al., 2014). However, in that study, PBP2a was detected in the total protein preparation. In the present study, for more precise detection of PBP2a in the membrane, membrane fraction was prepared following previously described protocol with slight modifications (Downer et al., 2002). Briefly, strains were grown in TSB overnight at 37°C with shaking at 200 rpm, then diluted 1/100 in 50 ml fresh medium and further incubated until cells reached an OD_600_ of 1. Ten milliliter of the bacterial cultures was then centrifuged, and the pellet was resuspended in 1 ml lysis buffer. Cells were disrupted by FastProtein^TM^ Blue Matrix using a FastPrep-24 homogenizer (MP Biomedicals) at 6 M/s for 4 cycles of 20 s with a 2-min ice incubation in between each cycle. Protein samples (1 μg of protein per lane) were separated in 10% sodium dodecyl sulfate polyacrylamide gel electrophoresis (SDS-PAGE), and then transferred to a polyvinylidene difluoride membrane (Immobilon, Millipore Corp., Bedford, MA, United States). PBP2a was probed with mouse monoclonal anti-PBP2a antibody (2F6F), and bands were visualized with HRP-conjugated secondary antibodies (Abcam Inc., Cambridge, MA, United States) followed by incubation in Western lightning chemiluminescence reagent plus (Perkin Elmer Life Sciences, Boston, MA, United States). Sortase A, a transpeptidase, was used as an internal control, identified by rabbit polyclonal anti-Sortase A primary antibody (Abcam Inc.).

### Real-Time Quantitative PCR Analysis for *mprF*, *vraSR*, and Cell Wall Synthesis-Related Genes

For RNA isolation, strains were grown overnight with shaking at 37°C in MHB and diluted with 5 ml fresh medium to 1/100. They were then grown at 37°C with shaking at 200 rpm, and samples were collected from four time points (2, 3, 4, 5 h) for analysis. Approximately 2 × 10^9^ cells were harvested for RNA isolation. Total RNA isolation was performed as described previously (Chen et al., 2009). Real-time quantitative PCR analysis was performed using Maxima SYBR Green qPCR Master Mix (Thermo Fisher Scientific, United States) on a Roche LightCycler 480 II Real-Time PCR System (Roche). Results were normalized to the expression of *gyrB*. The expression levels of *mprF*, *vraSR*, and several selected cell wall synthesis-related genes (*fmtA*, *murZ*, *pbp2*, *sgtB*, *tagA*) were determined using the primers listed in **Table 2**. The expression of *ldh1*, a gene involved in nitric oxide resistance in *S. aureus* (Richardson et al., 2008), was used as a negative control. The target gene transcripts were quantified by using the basic relative quantification method of the LightCycler 480 Software v1.5.1.62 (Roche). Three independent experiments were performed in duplicate and results are shown as mean ± SD. The mRNA expression levels of genes from CGK5 were defined as 1.

### Construction of *mprF* and *vraSR* Promoter-Reporter Plasmids

For introducing the pluxT2 reporter into CGK5, the selection marker *spc* was amplified by PCR from the Z172 strain (Chen et al., 2013), digested with SmaI and then cloned into pluxT2 (Chen et al., 2014) to generate pluxT2-SPC. The *mprF* and *vraSR* promoter fragments were PCR-amplified from the chromosome of CGK5 using the primers P*mprF*-F, P*mprF*-R and P*vraSR*-F, P*vraSR*-R. These amplified promoter fragments were fused upstream of the *lux* reporter genes of pluxT2-SPC using the BamHI and SalI sites, and the resulting plasmids were used for transformation of the *S. aureus* strain RN4220 and then of CGK5 and CGK5 mutant derivatives. The resulting promoter-reporter fusion constructs were confirmed by restriction enzyme analysis and DNA sequencing.

### Luciferase Reporter Assays

Bioluminescence was measured with a SpectraMax L microplate reader (Molecular Devices, Sunnyvale, CA, United States) to determine the transcriptional level of the *mprF* and *vraSR* promoter constructs. Three independent transformants harboring promoter-reporter fusion plasmids were grown overnight with shaking at 37°C in TSB containing spectinomycin for reporter plasmid maintenance, diluted 1:100 in the same medium, and aliquots (200 μl) of the cultures were transferred into 96-well plates in duplicate and incubated at 37°C. Both OD_595_ and bioluminescence (in relative light units, RLU) were monitored every hour for 7 h.

### Statistical Analysis

Assay results are reported as mean ± SD where appropriate. For comparison of differences between CGK5 and CGK5 mutant derivatives, the Student’s *t*-test was performed using GraphPad Prism 6 software (GraphPad, La Jolla, CA, United States). A *P* value less than 0.05 was considered statistically significant.

## Results

To examine the effects of the MprF L431F amino acid substitution in an isogenic background, we replaced the chromosomal wild-type *mprF* gene of the DAP-S parental strain CGK5 with the mutant *mprF* gene from the CGK6 DAP-NS strain, creating the new strain CKG5mut. A single base change (from CTT to TTT) causes the L431F mutation in MprF. Furthermore, we generated a reverse mutation complementation construct from CGK5mut, called CGK5mutR, which carries a wild-type *mprF* with a new EcoRV restriction site that can be used to differentiate CGK5mutR from CGK5. CGK5mut and CGK5mutR were verified by PCR and sequencing of the entire *mprF* gene. We then carried out a phenotypic analysis to compare CGK5mut and CGK5mutR against their parental strain CGK5.

**FIGURE 1:**
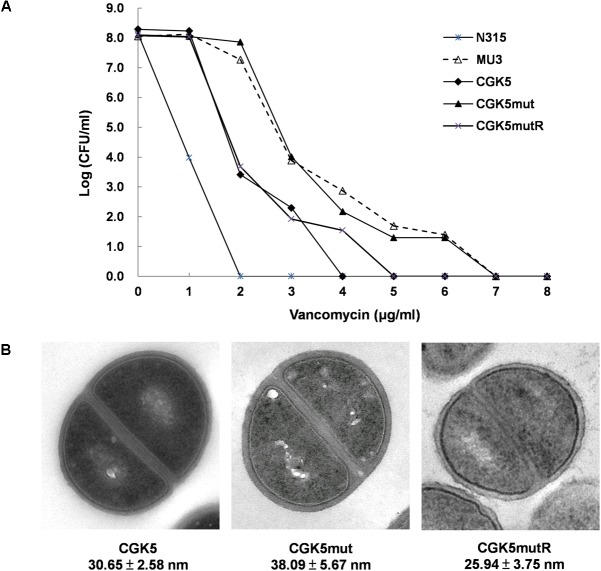
Detection of hVISA features. **(A)** Population analysis profile of vancomycin on CGK5 and its mutant derivatives. Overnight cultures of test isolates were serially diluted in Trypticase Soy broth (TSB) and inoculated onto brain heart infusion (BHI) agar plates containing 0 to 8 μg/ml of vancomycin. After 48 h incubation at 35°C, colonies were counted and the results were plotted on a graph. Mu3 (ATCC 700698) and N315 *S. aureus* strains were tested in parallel as hVISA positive and negative controls, respectively. **(B)** Transmission electron microscopy images of CGK5 and its mutant derivatives. Cell wall thickness (in nanometers) was measured at 150,000× magnification. Fifty measurements of equatorially cut cells were taken for calculation of cell wall thickness, and results are expressed as mean ± SD. CGK5, progenitor of CGK5mut; CGK5mut, derivatives of CGK5 with L431F mutation in *mprF*; CGK5mutR, reverse complementation construct of CGK5mut.

**FIGURE 2:**
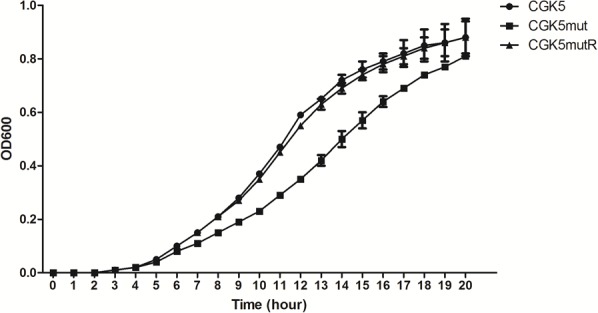
Growth curve of CGK5, CGK5mut, and CGK5mutR in Mueller Hinton II broth (MHB). Overnight fresh cultures of the bacteria were adjusted in 0.85% NaCl to 0.5 McFarland turbidity, then diluted 1:200 in MHB to obtain ∼5 × 10^5^ CFU/ml starting inoculum. The inoculum was dispensed at 120 μl per well in triplicates to a 100-well plate and incubated at 37°C in Bioscreen C MBR. The OD_600_ of each well was read every 30 min for 24 h. The average OD of the blank wells was subtracted from the average of the triplicate test wells at each time point and plotted.

The daptomycin MIC of CGK5mut was 6-fold higher (3 μg/ml) compared to that of the wild-type parental strain CGK5 (0.5 μg/ml), a level of increase similar to what we previously reported for DAP-NS clinical isolates (Lee et al., 2010). To our surprise, the vancomycin MIC of CGK5mut was also increased compared to CGK5 (3 μg/ml vs. 1.5 μg/ml). The reverse complementation construct, CGK5mutR, showed the same level of susceptibility as CGK5. PAP results showed that, of the three strains, only CGK5mut displayed a hVISA phenotype (**Figure 1A**). The bacterial feature that is often associated with DAP-NS and VISA or hVISA strains is increased cell wall thickness (Camargo et al., 2008; Mehta et al., 2012a). Transmission electron microscopy analysis revealed that CGK5mut has a significantly thicker cell wall (38.09 ± 5.67 nm) than CGK5 (30.65 ± 2.58 nm) and CGK5mutR (25.94 ± 3.75 nm) (*p* < 0.05) (**Figure 1B**). It has also been reported that clinical and *in vitro*-derived *S. aureus* strains exhibiting increased vancomycin MICs grow slower than their progenitors (Cui et al., 2003; Camargo et al., 2008). This phenomenon has been attributed to increased cell wall synthesis at a biological cost to the resistant strains (Cui et al., 2003). We also found that CGK5mut (doubling time = 240 ± 9.5 min) grows slower than both CGK5 (doubling time = 158 ± 3.5 min) and CGK5mutR (doubling time = 170 ± 4.2 min) in MHB (**Figure 2**).

**FIGURE 3:**
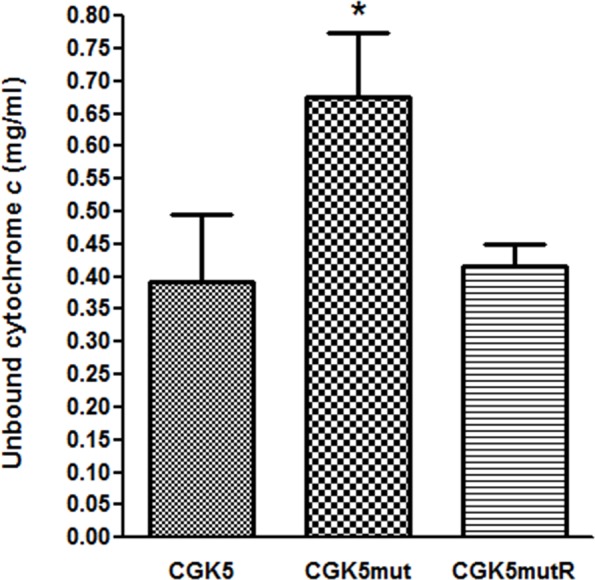
Binding of cationic cytochrome *c* to CGK5, CGK5mut, and CGK5mutR whole cells. The graph represents the unbound concentrations of cytochrome *c* after 10 min of incubation with CGK5 and CGK5 mutant derivatives. Results are expressed as mean ± SD from three independent experiments. ^∗^*p* < 0.05.

Altered cell membrane charge has been found in some DAP-NS *S. aureus* isolates with point mutations in *mprF* (Peschel et al., 2001; Oku et al., 2004). Cytochrome *c* is a cationic protein that has been used to estimate the relative bacterial cell surface charge of *S. aureus* (Mukhopadhyay et al., 2007). We detected a significant increase in unbound cytochrome *c* for CGK5mut (0.676 ± 0.056 mg/ml) compared to CGK5 (0.393 ± 0.058 mg/ml) and CGK5mutR (0.417 ± 0.02 mg/ml) (*p* < 0.05) (**Figure 3**), indicating an increased positive charge density on the cell surface of CGK5mut and suggesting a mechanism by which the L431F mutation of MprF might contribute to daptomycin resistance.

**FIGURE 4:**
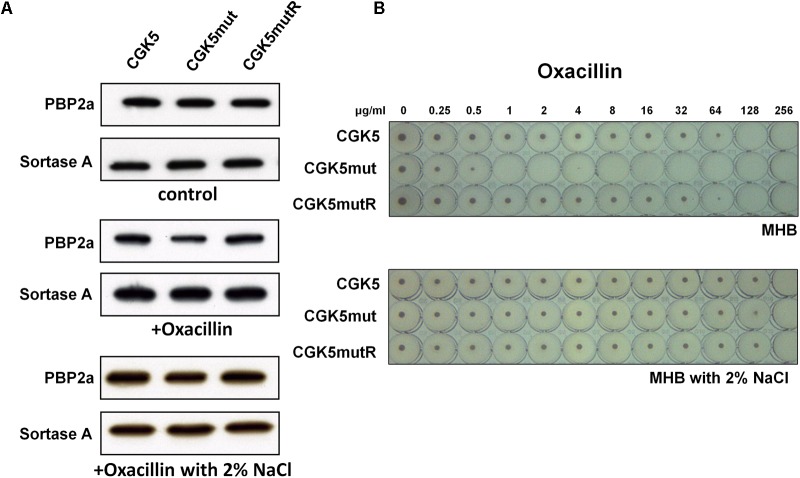
PBP2a production and the “seesaw-like” effect. **(A)** Production of penicillin-binding protein 2a (PBP2a) by CGK5 and CGK5 mutant derivatives was measured by Western blot analysis. Membrane protein was extracted from strains grown in medium alone (control) and in oxacillin (0.25 μg/ml)-containing medium with and without 2% NaCl. PBP2a was probed with mouse monoclonal anti-PBP2a antibody (2F6F), and sortase A was used as an internal control by using rabbit polyclonal anti-Sortase A primary antibody. **(B)** Oxacillin MICs of CGK5 and CGK5 mutant derivatives were determined by broth microdilution (BMD) in Mueller Hinton II broth (MHB; containing 2% NaCl or not) from an inoculum of 5 × 10^5^ CFU/ml.

The mechanism of oxacillin resistance in MRSA is thought to be mainly due to the production of penicillin-binding protein 2a (PBP2a, encoded by the *mecA* gene) with reduced affinity for β-lactams (Hiramatsu et al., 2001). Although first observed between glycopeptide and β-lactams (Sieradzki and Tomasz, 1999), the “seesaw” inverse relationship between daptomycin and β-lactam susceptibility has been reported by several groups (Mishra et al., 2009; Lee et al., 2010; Yang et al., 2010; Mehta et al., 2012a). We determined the level of PBP2a production in CGK5mut and found it to be comparable to that of CGK5 and CGK5mutR (**Figure 4A**).

However, concurrent to the increased daptomycin MIC, the oxacillin MIC of CGK5mut decreased to 1 μg/ml from 128 μg/ml in CGK5, as determined by the BMD method in medium without 2% NaCl, whereas CGK5mutR showed a similar MIC as CGK5 (**Figure 4B**). Thus, CGK5mut exhibits the “seesaw-like” effect similar to which we and others have previously observed in DAP-NS *in vitro*-selected and clinical *S. aureus* isolates (Lee et al., 2010; Mehta et al., 2012a,b). Interestingly, the oxacillin MICs of CGK5, CGK5mut and CGK5mutR were the same (256 μg/ml) in medium containing 2% NaCl (**Figure 4B**). Therefore, we further measured the level of PBP2a of these strains grown in oxacillin (0.25 μg/ml) containing medium with and without 2% NaCl. The level of PBP2a of CGK5mut showed a marked reduction after exposure to oxacillin, and 2% NaCl counteracted this effect (**Figure 4A**).

**FIGURE 5:**
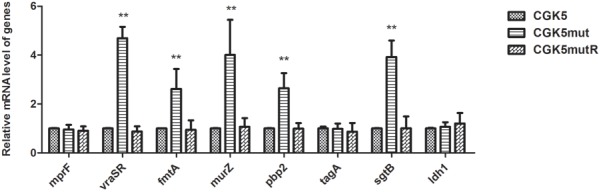
Transcriptional expression of *mprF* and *vraSR* and cell wall synthesis*-*related genes. Expression of *mprF* and *vraSR*-related genes in CGK5 and CGK5 mutant derivatives was compared by real-time quantitative PCR analysis. Three independent experiments were performed in duplicate at four time points (2, 3, 4, 5 h) and results at the 4-h time point are shown here as mean ± SD. The expression of *ldh1* was used as a negative control. The mRNA expression level of genes from CGK5 was defined as 1. ^∗∗^*p* < 0.01 when compared to CGK5.

Mutation of *mprF* has been shown to impact the expression of *mprF* itself (Yang et al., 2009b). In addition, VraSR, a two-component regulatory system that plays a major role in cell wall synthesis and is a key player in the cell-wall stress response, has often been implicated in vancomycin and daptomycin resistance (Kuroda et al., 2003; Camargo et al., 2008; Muthaiyan et al., 2008; Mehta et al., 2012a). We therefore compared the expression of *mprF* and *vraSR*-related genes in CGK5 and its mutant derivatives by real-time quantitative PCR analysis. We found there was no significant difference in the *mprF* expression among them (**Figure 5**). However, significant increases (>4-fold increase, *P* < 0.01) in the expression of *vraSR* were seen in CGK5mut compared to CGK5 and CGK5mutR at the 4-h time point. In addition, the expression levels of four examined cell wall synthesis-related genes (*fmtA*, *murZ*, *pbp2*, *sgtB*) were also significantly increased in CGK5mut (>2∼3-fold increase, *P* < 0.01) at this time point.

**FIGURE 6:**
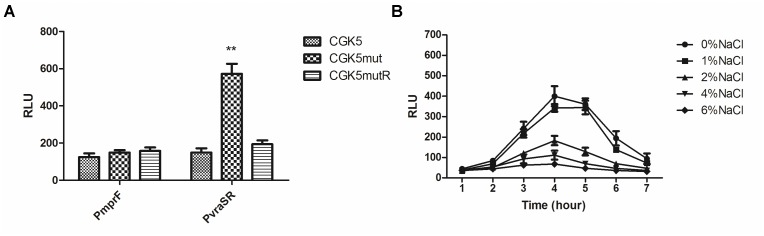
Transcriptional activity of promoters of CGK5 and its mutant derivatives. Bioluminescence assay was performed to determine the transcriptional level of the **(A)** *mprF* or *vraSR* promoter in CGK5 and CGK5 mutant derivatives (maximal activity at the 4-h time point is shown) and the effect of **(B)** salt concentration on *vraSR* promoter activity in CGK5mut. Data are expressed as relative light units (RLU) from three independent transformants tested in duplicate.

To clarify whether the up-regulation of gene expression in CGK5mut is due to promoter activity, we constructed *mprF* and *vraSR* promoter-reporters. The transcriptional activity of the *mprF* and *vraSR* promoters was compared in CGK5 and its mutant derivatives by using a bioluminescence assay. *mprF* promoter activity among the tested strains showed no obvious differences (**Figure 6A**). However, the *vraSR* promoter showed significantly higher promoter activity in CGK5mut compared to the other two strains and the maximum discrepancy was at the 4-h time point (**Figure 6A**), indicating that the up-expression of *vraSR* in CGK5mut was mediated by its promoter.

To determine whether the reduced oxacillin MIC of CGK5mut was caused by increased expression of *vraSR*, and how this might be related to the fact that 2% NaCl could restore the oxacillin MIC of CGK5mut to wild-type levels, we examined whether the high salt concentration could inhibit the expression of *vraSR* in CGK5mut. A *vraSR* promoter activity assay showed that NaCl does indeed inhibit *vraSR* promoter activity (**Figure 6B**). The effect is dose-dependent and 2% NaCl is the critical concentration for inhibition.

## Discussion

Daptomycin is an important last-line agent against serious *S. aureus* infection, particularly in patients with persistent MRSA bacteremia and endocarditis (Boucher and Sakoulas, 2007; Marco et al., 2008). Daptomycin non-susceptibility in *S. aureus*, albeit still rare, can emerge during treatment and usually in patients with severe deep-seated infections (Julian et al., 2007; Lee et al., 2010; Boyle-Vavra et al., 2011). The mechanism of daptomycin-non-susceptibility is still not fully understood but has been associated with multiple genetic changes on the bacterial chromosome, with single point mutations in the *mprF* gene being the most frequently identified alteration (Friedman et al., 2006; Julian et al., 2007; Mehta et al., 2012a; Mishra et al., 2012). Although a few hot spots have been associated with daptomycin resistance, such as L826F, S295L, and S337L, there is considerable variation in the locations of the mutations in the MprF polypeptide structure (Friedman et al., 2006; Julian et al., 2007; Yang et al., 2009a; Lee et al., 2010; Boyle-Vavra et al., 2011; Mehta et al., 2012a; Peleg et al., 2012). A study just published by Yang et al. (2018) investigated the impact of laboratory generated single or dual point mutations within the hot spot loci of *mprF* and found that extra point mutation in *mprF* resulted in diminished characteristics associated with DAP-NS, which may explain why no clinically derived DAP-NS strains contained multiple point mutations within the *mprF* gene.

Our previous study on sequential clinical MRSA isolates from a patient with persistent bacteremia revealed an L431F amino acid substitution in the 3 DAP-NS isolates we characterized (Lee et al., 2010). Since this mutation had not been seen in other DAP-NS isolates reported to date, we carried out the present study to verify its contribution to daptomycin resistance. Because some DAP-NS isolates are also vancomycin non-susceptible, and a few DAP-NS isolates (including our own; Mishra et al., 2009; Lee et al., 2010; Yang et al., 2010; Mehta et al., 2012a) have been reported to exhibit a reduced oxacillin MIC, we also investigated the effects of this L431F MprF change on vancomycin and oxacillin MICs. The approach we employed differed from those of other studies in that we used a single-base-substitution (from CTT to TTT) method to create the L431F mutant (CGK5mut) from CGK5, the wild-type progenitor of our DAP-NS strains. We also generated a reverse mutant from CGK5mut, CGK5mutR, to verify our findings for CGK5mut. This approach ensured that the strains we tested and compared were isogenic, except for the single nucleotide difference at the site corresponding to codon 431 of MprF (and a mutated silent EcoRV site in CGKmutR which has no effect on the sequence of the polypeptide).

The increased daptomycin MIC observed in CGK5mut confirmed that the L431F missense mutation contributes to daptomycin resistance. We also showed that CGK5mut has reduced binding of cytochrome *c*. Since the L431 amino acid is located in the C-terminal LPG synthase domain of MprF (Ernst et al., 2009), the daptomycin-non-susceptibility in CGK5mut might be an effect of a reduced PG:LPG ratio (Ernst et al., 2009; Yang et al., 2009b). A lower PG:LPG ratio has been shown to reduce the negative charge of the cell membrane (Baltz, 2009; Rubio et al., 2012), thus diminishing the access of calcium-dependent daptomycin to its cellular target.

Another factor that is commonly associated with daptomycin-non-susceptibility is VraSR (Camargo et al., 2008; Mehta et al., 2012a), a two-component system that positively regulates the cell wall biosynthesis pathway and is involved in the cell wall stimulon (Kuroda et al., 2003; Gardete et al., 2006). The VraSR system was originally identified as a key regulator of vancomycin resistance in VISA and hVISA isolates (Kuroda et al., 2000; Cui et al., 2005). We found that the expression levels of *vraSR* and several *vraSR*-regulated genes were significantly increased in CGK5mut compared to CGK5 and CGK5mutR. In addition to the increased daptomycin MICs, the vancomycin MIC of CGK5mut was also increased, and population analysis showed that more CGK5mut isolates were able to grow in higher concentrations of vancomycin compared to CGK5 and CGK5mutR. CGK5mut cells also have a thicker cell wall and grow slower than CGK5 and CGK5mutR cells. These cellular features have been associated with both DAP-NS *S. aureus* and hVISA/VISA (Camargo et al., 2008; Song et al., 2013). The changes in CGK5mut vancomycin MIC and cell wall thickness likely arise from the upregulated *vraSR*-dependent system. But how the L431F mutant protein brings about the increased expression of *vraSR* and the cell wall-related genes is unknown. However, CGK5mut had significant increased *vraSR* promoter activity compared to CGK5 and CGK5mutR using *vraSR* promoter-reporter assay, indicating that the up-expression of *vraSR* in CGK5mut was mediated by its promoter. A previous study observed the mutual presence of both *mprF* point mutations and increased expression levels of *vraSR* in DAP-NS strains compared to their DAP-susceptible counterparts, and hypothesized that both genes were mechanistically linked to the DAP-NS phenotype (Mehta et al., 2012a). However, our results suggest a causal relationship between *mprF* point mutation and increased expression of *vraSR*, which may explain why the daptomycin resistance is often concomitant with vancomycin resistance in clinical isolates. There are a plethora of pathways to heterogeneous and intermediate resistance to vancomycin in *S. aureus* (Howden et al., 2010). In addition to VraSR, two other two-component systems, GraSR and WalKR, have also been associated with reduced susceptibility of *S. aureus* to both daptomycin and vancomycin. It has been reported that GraSR also plays an important role in *S. aureus* resistance to cationic antimicrobial peptides via altered expression of *mprF* and *dltABCD* resulting in increased electrostatic repulsion of cationic antimicrobial peptides. GraSR has also been shown to interact with the WalKR system and have significant regulatory overlap (Li et al., 2007; Meehl et al., 2007; Dubrac et al., 2008; Falord et al., 2011). However, we found no significant differences in the expression of *mprF* and *mprF* promoter activity among the tested strains. Therefore, it appears unlikely that reduced susceptibility of CGK5mut to both daptomycin and vancomycin are associated with these two two-component systems.

Although expression of PBP2a (encoded by the *mecA* gene) is thought to play a major role in oxacillin-resistance, several studies have reported that the amount of PBP2a expressed does not correlate with the level of methicillin-resistance (Chambers and Hackbarth, 1987; Murakami and Tomasz, 1989; De Lencastre et al., 1994), and other auxiliary genes have been reported to be essential for the optimal expression of methicillin-resistance (De Lencastre and Tomasz, 1994). When we determined the production level of PBP2a in CGK5mut and found it to be comparable to that of CGK5 and CGK5mutR in the normal condition, we thought its “seesaw-like” effect may be caused by other factors. This indicated that the expression of *mecA* gene in CGK5mut was not altered by the *mprF* point mutation. Since the oxacillin MIC of CGK5mut was reduced in medium lacking 2% NaCl, we measured the levels of PBP2a in these strains in the presence of oxacillin with and without 2% NaCl and found that the level of PBP2a production in CGK5mut reduced after exposure to oxacillin and 2% NaCl could abolish this effect. According to the CLSI guideline, 2% NaCl should be added to the medium in determining the oxacillin MIC for staphylococci (CLSI, 2013). Interestingly, our results also showed that the expression of *vraSR* decreased in the presence of 2% NaCl, with a concurrent restoration of oxacillin resistance in CGK5mut, indicating that the effect of upregulation of *vraSR* in CGK5mut could be abolished by adding 2% NaCl. Since the “seesaw” effect has been observed in only a few DAP-NS MRSA isolates (Mehta et al., 2012b), the factors associated with this phenomenon warrant further investigation. However, *mprF* point mutation in CGK5mut only exhibits the “seesaw-like” effect (meaning the seesaw effect was only observed in the absence of NaCl), we speculate that additional mutations at other genes are required to achieve truly “seesaw” effect observed in clinical isolates. In addition, how 2% NaCl counteracts the up-regulation effect of *vraSR* caused by MprF L431F mutation requires further research.

In fact, Mehta et al. (2012a) previously observed the phenotype in DAP-non-susceptible strains with upregulation of the two-component regulatory system *vraSR*; however, they did not prove this was caused by the MprF mutation. By contrast, they introduced an overexpression of *vraSR* in DAP-susceptible strains to show a reduction in oxacillin resistance. In their latest study, they found that introducing an overexpression of *vraSR* in DAP-susceptible strains triggering MprF mutation and result in impairment of PrsA chaperone functions, both events are required for β-lactam resistance via PBP2a maturation (Renzoni et al., 2017). It has long been recommended that 2% NaCl (or KCl) be added to the culture medium to reliably detect oxacillin resistance in staphylococci (Thornsberry and McDougal, 1983; Huang et al., 1993). When salt is placed in solvent, the solid salt dissolves into its component ions, sodium (Na^+^), potassium (K^+^), chloride (Cl^-^), which are the primary ions of electrolytes in physiology. It is plausible to speculate that these ions maintain the phosphorylation state of VraS in respond to *mprF* point mutation and/or β-lactam stimulon to precisely regulate its downstream genes for the generation of resistant phenotype.

## Conclusion

The present study demonstrated that a single amino acid substitution (L431F) in the MprF protein contributes to both daptomycin and vancomycin resistance as well as increased oxacillin susceptibility in an isogenic MRSA strain. This point mutation in CGK5 also causes up-expression of *vraSR*. Further studies on the mechanisms contributing to these phenomena may lead to discovery of potential therapeutics against multidrug-resistant staphylococci.

## Author Contributions

F-JC contributed to the study design, data analysis, and manuscript preparation. T-LL performed the data analysis and prepared the manuscript. C-HL, Y-CH, I-WH, P-CH, and C-SY performed the work and data analysis.

## Conflict of Interest Statement

The authors declare that the research was conducted in the absence of any commercial or financial relationships that could be construed as a potential conflict of interest.

## Footnotes

1 http://emboss.bioinformatics.nl/cgi-bin/emboss/silent
